# Assessing the delivery of smoking cessation interventions in adult inpatients

**DOI:** 10.1192/bjo.2021.822

**Published:** 2021-06-18

**Authors:** Katie Blissard Barnes, Richard Westmoreland

**Affiliations:** LYPFT NHSFT

## Abstract

**Aims:**

To assess level of compliance with national and local guidance with regards to the recording of service users smoking status and offering of interventions.

**Background:**

Across the general population, prevalence of smoking is decreasing but in those with severe mental illness, the prevalence hasn't significantly changed. LYPFT are working towards becoming a smoke-free trust. The Trust Guidance expects that Trusts should ask 100% of service users if they smoke (which should be recorded on their physical health CQUIN) and of those that do, should be offered nicotine replacement therapy and cessation advice. Public Health England is working towards all hospital trusts across the UK being Smoke-free.

**Method:**

All service users on each of the 4 adult inpatient wards at the Becklin Centre, Leeds, were included in the audit. A total of 78 service users were included in the audit.

We reviewed the digital records for every service user, specifically looking at the physical health CQUIN. We recorded if smoking status had been documented and what interventions (if any) had been recorded as given. Possible interventions included offering brief advice and offering Nicotine replacement therapy. We then reviewed medication charts to see if any nicotine replacement therapy had been prescribed.

**Result:**

The audit found that approximately half of all service users in our audit smoked cigarettes and that the vast majority of these had their smoking status documented in their digital medical records.

Three quarters of those that smoked were offered brief cessation advice and half of them were offered Nicotine Replacement Therapy. Only a third of service users that smoked had NRT prescribed on their medication chart. This represented 65% of those recorded as being offered NRT.

**Conclusion:**

There are numerous possible reasons for the above outcomes. These include a lack of knowledge and confidence in delivering smoking cessation interventions, conversations having taken place but not recorded and confusion regarding the appropriate staff member to deliver the intervention. In addition, whilst only medical professionals typically prescribe NRT, the physical health CQUIN is recorded by nurses. Therefore, this may reflect a lack of communication between staff groups.

Our trust will become smoke free in the near future. To facilitate this, we hope to reduce the discrepancy between the number of service users who smoke and the number prescribed NRT.

